# On the use of complexity algorithms: a cautionary lesson from climate research

**DOI:** 10.1038/s41598-020-61731-7

**Published:** 2020-03-19

**Authors:** Alfonso Delgado-Bonal

**Affiliations:** 1 NASA Goddard Space Flight Center, Earth Sciences Division, Greenbelt, Maryland USA; 2 Universities Space Research Association, Columbia, Maryland USA

**Keywords:** Climate change, Statistical physics, thermodynamics and nonlinear dynamics

**arising from**: Z. Shao, *Scientific Reports* 10.1038/s41598-017-04584-x (2017).

## Introduction

Complexity algorithms provide information about datasets which is radically different from classical moment statistics. Instead of focusing on the divergences from central values, they quantify other characteristics such as order, pattern repetitions, or the existence of attractors. However, those analyses must be done with the proper statistical treatment, which is, unfortunately, not always the case. In this contribution, I provide an example of the hazards of applying complexity measures without sufficient care by correcting a previously published analysis that aimed to quantify the complexity of climate. I clarify some misconceptions about the use of Sample Entropy and revise the incorrect assessments and conclusions drawn from the previous misapplication of the methods.

An attempt to contrast the complexity of the climate of the past 122,000 years and the recent 2,000 years has been developed recently^1^. The idea behind the research was to use the Oxygen isotopic (*δ*^18^*O*) record of ice cores, a proxy for the temperature at the accumulation site through history, to determine the levels of complexity of the two different periods. The author obtained values of Sample Entropy (SampEn) of 0.7  ±  0.1 for the long record and 2.2  ±  0.2 for the short one, and values of Lempel-Ziv complexity (LZC) of 0.29  ±  0.03 and 0.99  ±  0.05 for the long and short records respectively, attributing those differences to changes in the complexity of climate. In this paper, I use the same data to show that the reported differences are attributable to the incorrect use of the algorithms rather than to actual changes in climatic complexity.

Complexity algorithms are statistical tools, and as such, the conclusions drawn from them are as good as the robustness of their application. The first issue with the previous research was related to the use of the data. The author used two different datasets of the *δ*^18^*O* record with different time resolution: the data record for the last 2,000 years had a time resolution of *τ* = 1 year, while the record for 122,000 years had a time resolution of *τ* = 20 years. The complexity algorithms used in this research require measurements equally spaced in time^2^,^3^ and, in general, the comparison of different time resolutions is impractical and lacks any statistical significance. In the second figure of the same research, the author also determined the complexity for the recent past 2,000 years by sampling the annually resolved record every *τ* = 20 years, obtaining a mean value of SampEn = 2.3  ±  0.5 and LZC = 1.16  ±  0.08. However, the NGRIP *δ*^18^*O* data set spanning the entire past 122,000 years consists of 20-year binned averages of the higher-resolution measurements. In order to avoid erroneous conclusions, instead, this data should have been compared to 20-year binned averages of the annually resolved record.

The second issue with the previous research was the misapplication of the complexity algorithms. The complexity of climate was determined by making use of Sample Entropy^4^ and Lempel-Ziv complexity^5^.

SampEn was designed as a variation of Approximate Entropy (ApEn)^2^. These algorithms give higher values to complex and random systems and lower values to more predictable series. Both ApEn and SampEn are based on the probability of occurrence of patterns of data and have proved their validity to distinguish normal from abnormal data in instances where moment statistic approaches failed to show significant differences^3^. Furthermore, both statistics are relative measures and comparisons are intended between data series of the *same length* with *homogeneous* generating processes.

Lamentably, it is generally assumed that “SampEn is largely independent of record length”^4^. That idea, introduced in the literature by the creators of Sample Entropy, is extremely misleading and requires a clarification: in the case of a homogeneous data generating process, SampEn is independent on the record length whereas ApEn is biased in that sense. If the generating process does not change, there is a similar frequency of occurrence of a particular pattern and SampEn could be used independently of the number of observations while ApEn would require a large number of observations to be accurate. However, as evidenced in the next section, climate has changed dramatically during the last 122,000 years mixing *heterogeneous* epochs, and the results of the algorithm depend on the length of the data record. A comprehensive tutorial and detailed analysis of the SampEn and ApEn equations can be found in^6^.

**Figure 1:**
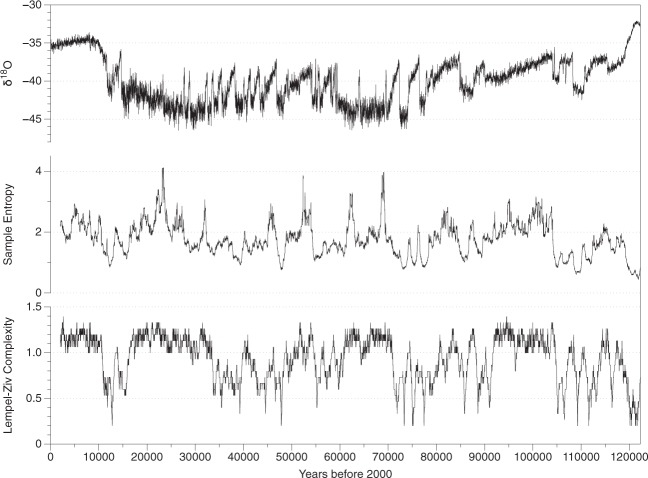
Top: *δ* ^18^*O* record of ice cores (years before 2000) for the last 122,000 years. Center: Sample Entropy rolling windows of 2,000 years length. Bottom: Lempel-Ziv Complexity rolling windows of 2,000 years length.

Similarly, the comparisons of Lempel-Ziv complexity (LZC) lack of validity since they are made between heterogeneous epochs with a different number of data. In essence, this algorithm transforms the data record into a binary chain depending on the different values being higher or lower than the median, and then the number of patterns is analyzed. Given the highly variable shape of the original *δ*^18^*O* record from Fig. 1 (top), the comparison of each value with the median (corresponding to  −39.88) is translated into a binary chain of many ones or zeros in a row, indicating low complexity for the 122,000 years dataset. On the contrary, as the 2,000 years record is almost flat, the mean divides the string into ones and zeros more diversely, providing more complexity and randomness.

In the next section, I show that throughout the last 122,000 years we can find subsequences of 2,000 years with values of SampEn and LZC similar to the most recent 2,000 years. This indicates that the previous 2,000 years do not stand out in terms of the complexity estimated from the *δ*^18^*O* record. Moreover, the results of the last 2,000 years are close to the mean values of the analysis. Therefore, the claim that the whole 122,000 years record is less complex than the last 2,000 years is unjustified and is nothing more than a statistical artifact.

## Results

I analyze here three important issues arising when applying complexity algorithms: (*i*) the mixing of heterogeneous epochs; (*ii*) the length of the sequences; (*iii*) and the non-stationarity of the data.

(*i*) Both ApEn and SampEn have their roots in the entropy concept from Information Theory, where the alphabet is the list of possible outcomes of a random variable. In an experiment with dice, the alphabet would be the integers from 1 to 6. If we change that dice for a 12-sided dice, the alphabet would change and the comparison with the previous data record would be inaccurate; the frequency of occurrence of the different numbers has changed radically. The same situation happens in the analysis of climate. Figure 1 (top) shows that the *δ*^18^*O* data changed suddenly around 11,700 years ago, corresponding to the beginning of the Holocene and the abrupt warming after the last glacial period. The assumption that the epochs before and after that event have the same alphabet may lead to incorrect comparisons since both Shannon (for ApEn) and Rényi (for SampEn) entropies are dependent on the alphabet.

The goal of complexity algorithms is to characterize changes in similar data series. In this sense, we shall remember that both algorithms were designed for clinical applications and they have been used to analyze, for example, the changes in the complexity of heartbeats. The comparison of the results must be made, continuing with the example, between two time series of human heartbeats since they have similar alphabets; the direct comparison of the complexity of the heartbeat of two different species would not provide meaningful information. For an application of complexity measures to series with different alphabets, see^7^.

In Fig. 1, the LZ complexity roughly indicates how many regime shifts there are since the algorithm compares each value with the mean. Thus, Holocene values correspond to values during the glacial where there are almost no abrupt transitions.

(*ii*) Using sequences of the same length is fundamental and the comparison of 2,000 years with 122,000 years is impractical and leads to misleading results. To show that, I analyze the changes of complexity through time using rolling windows of 2,000 years. If the complexity estimates for the last 2,000 years were substantially different, it should stand out of the range of values obtained in all other windows of the same length.

Instead of using two different time series, I have made the calculations using only the NGRIP *δ*^18^*O* record of the entire previous 122,000 years on the GICC05modelext time scale in 20-year resolution. This record spans from 20 to 122,280 years b2k (before the year 2000) and contains N = 6114 observations, including the last 2,000 years. For the analysis, I divide the whole record into sequences of 2,000 years: the first sequence contains the data from [20, 2000] years b2k, the second sequence the data from [40, 2020] years b2k, etc. The time resolution is always *τ* = 20 years and the length record is *N* = 100 for all sequences.

Figure 1  shows the whole *δ*^18^*O* record (top), the Sample Entropy for the different sequences of 2,000 years (center), and the Lempel-Ziv complexity for the different sequences of 2,000 years (bottom). The mean value of SampEn for all the sequences is 1.76, and the standard deviation is 0.56. The mean value of LZC for the same sequences is 0.93, and the standard deviation is 0.24. The uncertainty in ApEn and SampEn is low; the standard deviation of ApEn(2, 0.15*σ*, 1000) determined through Monte Carlo simulations is less than 0.055 for a large class of models^9^.

It is clear from the figure that the first sequence corresponding to the recent past 2,000 years is not substantially different from the rest of the sequences, having a SampEn value of 2.18 and a LZC of 1.20. Therefore, there is no scientific argument to justify the claim that “By comparison, the recent 2000-year climate is further dominated anthropogenic forcing processes besides natural forcing processes”^1^. Furthermore, previous epochs have had higher levels of complexity with values of SampEn around 4, four times the standard deviation from the mean. In those epochs, however, only natural forcing processes can be claimed.

**Figure 2:**
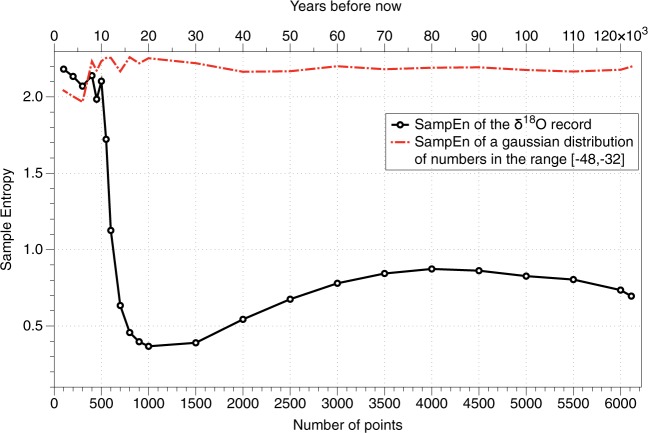
Dependence of SampEn with the number of points considered for the *δ*^18^*O* GICC05 record (black) and for a homogeneous gaussian dataset with random numbers distributed in the range [−48, −32] (red).

As stated above, a general misconception about SampEn is that it is said to be independent on the record length^4^. In Fig. 1 (top) it can be seen that the climate has been very heterogeneous, and displayed a *δ*^18^*O* minimum of  − 46.5 and a maximum of  −32. To show that SampEn is not always independent of the record length, I generate a homogeneous gaussian set of random numbers contained between [−46.5, −32] and study the dependence of SampEn with the number of points considered. Figure 2 (red line) shows the results of that analysis. It can be seen that the SampEn of the homogeneous gaussian set behaves almost independently of the number of points considered. Therefore, it would be possible to compare a sequence of 1000 points with a sequence of 6000 points for that dataset. However, as the real *δ*^18^*O* contains trends and changes in variance, SampEn is strongly dependent on the number of points (black line). If instead of analyzing 2000 years (100 points) of data like in^1^ we would consider 10,000 years (500 points) the complexity of the data would be similar, but as soon as data older than 12,000 years is included (600 points), SampEn drops dramatically because of the first regime-shift appearing (deglaciation from the glacial period to the Holocene). The tolerance of SampEn is measured as a function of the standard deviation, and abrupt variations in the data will change the sensitivity of complexity measures, making the results dependent on the number of points for heterogeneous datasets.

The effects of a small sample dataset are evidenced in the gaussian set (red line) for a number of points lower than 300.

(*iii*) Finally, I address the issue of the non-stationary of the data. Before doing the analysis, the stationarity of the dataset can be studied with unit root tests such as Dickey-Fuller or Kwiatkowski–Phillips–Schmidt–Shin (KPSS). The non-stationarity of the *δ*^18^*O* record implies, for example, that the mean of the whole 122,000 years record (−39.94) is substantially different from the mean of the first subsequence of 2,000 years (−35.32). Following Pincus, who designed the ApEn algorithm in the first place^8^, “If the time series is nonstationary, that is, contains one or more pronounced trends upward or downward, little can be inferred from moment (mean, variability), ApEn, or power spectral calculations, because the trends tend to dominate all other features. Physiologically, data with trends suggest a collection of heterogeneous epochs as opposed to a single homogeneous state. From the statistical perspective, it is imperative that any trends be removed before meaningful interpretation can be made from the statistical calculations”.

**Figure 3:**
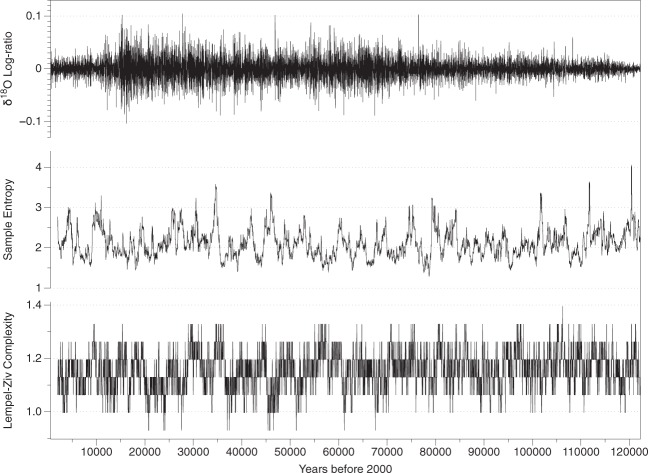
Top: Stationary log-ratio *δ*^18^*O* record of ice cores for the last 122,000 years. Center: Sample Entropy rolling windows of 2,000 years length for the stationary sequences (*N* = 100) with mean and standard deviation of 2.110  ±  0.353. Bottom: Lempel-Ziv Complexity rolling windows of 2,000 years length for the stationary sequences (*N* = 100) mean and standard deviation of 1.156  ±  0.073.

To properly quantify the complexity of the dataset, I transform the *δ*^18^*O* record by taking logarithms to stabilize the variance and then subtracting the previous value of the ${\rm{\log }}\,$ to eliminate the trend (log-ratio transformation, ${\rm{\log }}\,({x}_{t}/{x}_{t-1})$)^9^. The augmented Dickey-Fuller and KPSS tests verify that the transformed data set is stationary, and therefore the algorithms can be used to quantify complexity. Figure 3 (top) shows the variability of the *δ*^18^*O* record along with the measures of complexity (center and bottom). The mean and standard deviation of SampEn is 2.11  ±  0.35 and of LZC is 1.16  ±  0.07. It is clear from those figures that the last 2,000 years do not have any particularity regarding its complexity, because they are comparable to many other sequences of similar length throughout the entire last glacial period.

## Data and Methods

This research uses the *δ*^18^*O* record of the NGRIP ice cores provided by the Center of Ice and Climate of the University of Copenhagen, the same dataset used in the original research^1^. In particular, the data are those defined as: "Greenland Ice Core Chronology 2005 (GICC05) time scale for the NGRIP ice core (down to 60 ka b2k) extended to the base of the NGRIP ice core by merging with the ss09sea06bm model time scale. The resulting time scale is called GIC05modelext. Also supplied are 20 year means of *δ*18O data from NGRIP back to 123 ka b2k/3085 m depth. “The dataset is freely accessible at http://www.iceandclimate.nbi.ku.dk/data  and described in^10–14^.

The methods used in this paper are also those described in the original research.

## Conclusions

In this paper, I reanalyzed the complexity of the last 122,000 years using the *δ*^18^*O* record of the NGRIP ice core to check the validity of the claims made in^1^. I explained some of the major misconceptions when applying complexity measures and specifically showed that SampEn is not independent of the length of the dataset in general situations.

To show that the previously published analysis was inaccurate, instead of only comparing the last 2,000 years with the previous 122,000 years, I examined all consecutive sequences of 2,000 years, indicating that the complexity of the recent climate does not stand out from earlier segments of the Holocene or last glacial period. The values obtained for the recent past are within one standard deviation from the mean.

By making proper use of the algorithms, I showed how different epochs have had varying levels of complexity and some periods stand out by having a SampEn above 3 times the standard deviation from the mean. The ability to specify which epochs had greater complexity is useful for paleoclimate research to investigate the causes behind it, providing helpful knowledge to the geophysical community. However, based on the results of this paper, the claim that anthropogenic forcing processes have changed the complexity of the current climate in the last 2,000 years measured by the *δ*^18^*O* record up to a level which is different from previous epochs seems to be unjustified.

Complexity algorithms are an excellent tool to obtain insights about nonlinear dynamic processes and characterize the changes in a system. However, as in any other statistical analysis, it is essential to make a reasonable use of the algorithms by checking that we are not mixing heterogeneous epochs, verifying the effect of the length of the sequences, and studying the stationarity of the data. In general, the algorithms should be used with stationary data. However, it is not uncommon to find applications of ApEn and SampEn to raw data: in those instances, the comparisons should be made between similar systems with the same kind of cycles and trends.

## Data Availability

This research uses the *δ*^18^*O* record of the NGRIP ice core provided by the Center of Ice and Climate of the University of Copenhagen, freely accessible at http://www.iceandclimate.nbi.ku.dk/data.
